# Correction to: Perforated anterior tenia coli-type appendicitis in a case of vermiform appendix duplex in a toddler: a case report

**DOI:** 10.1093/jscr/rjaf521

**Published:** 2025-07-24

This is a correction to: Zlatan Zvizdic, Melika Bukvic, Nermina Ibisevic, Alena Firdus, Asmir Jonuzi, Semir Vranic, Perforated anterior tenia coli-type appendicitis in a case of vermiform appendix duplex in a toddler: a case report, *Journal of Surgical Case Reports*, Volume 2024, Issue 12, December 2024, rjae784, https://doi.org/10.1093/jscr/rjae784

The originally published version incorrectly stated that Open Access publication was funded by the Qatar National Library. That statement has been removed.

